# Can arthrocentesis help reduce the displaced disc in patients with closed lock of the temporomandibular joint? A case review

**DOI:** 10.4317/jced.62613

**Published:** 2025-04-01

**Authors:** Jordi Borrás-Ferreres, Cosme Gay-Escoda

**Affiliations:** 1 DDS. MSc. Private practice of Oral Surgery and Implantology, Benicarló (Castellón), Spain; 2 MD, DDS, MSc, PhD, EBOS, OMFS. Chairman and Professor of the Department of Oral and Maxillofacial Surgery, Faculty of Medicine and Health Sciences, School of Dentistry, University of Barcelona. Director of the Master degree program in Oral Surgery and Implantology, EFHRE International University/FUCSO, Barcelona. Founder/Researcher of the IDIBELL Institute. Head of the Department of Oral and Maxillofacial Surgery and Implantology, Teknon Medical Center, Barcelona, Spain

## Abstract

Arthrocentesis (joint lavage and lysis) and hydraulic distension of the temporomandibular joint have been described as effective options for reducing joint pain and improving function in patients with limited mouth opening (closed lock) due to disc displacement without reduction, fundamentally in the acute phase of the disorder. Despite controversy, some studies suggest that in addition to improving the range of opening and reducing joint pain, the disc can also be reduced in some cases. The present study was carried out to determine whether arthrocentesis could reduce the displaced disc in a woman with 6 weeks of closed lock, and to assess its efficacy in improving mouth opening and reducing joint pain.

** Key words:**Arthrocentesis, closed lock, disc displacement, limited mouth opening, disc reduction, disc recapture.

## Introduction

Closed lock of the temporomandibular joint (TMJ) is the result of a usually anterior or anteromedially displaced joint disc that is not reducible and acts as an obstacle against anterior displacement of the mandibular condyle (1). The clinical signs of this disorder are restricted anterior displacement of the mandible, the absence of joint sounds (clicks), deflection towards the affected side on opening the mouth, limitation of lateral motion towards the contralateral side, and the restriction of protrusion movements, with mandibular displacement towards the affected side. In acute cases, joint pain is noted in response to palpation and during opening of the mouth (2). The diagnostic imaging technique of choice is magnetic resonance imaging (MRI), where closed lock is seen as disc displacement without reduction (DDwoR) with the mouth open (1,3).

Arthrocentesis (joint lavage and lysis) is used to cleanse and break up small adherences in the superior joint space (SJS), and hydraulic distension of the TMJ in turn aims to insufflate the SJS to reduce the intra-articular pressure. Both techniques have been found to be effective in reducing joint pain and in increasing the range of mouth opening in patients with closed lock of the TMJ due to DDwoR (4,5). The reported success rate of arthrocentesis in closed lock of the TMJ ranges between 70-95% (6,7). However, could simple lavage, lysis and hydraulic distension of the SJS contribute to reduce the joint disc during mouth opening? Some studies suggest that the morphological and organic changes of the TMJ with DDwoR are so severe that disc reduction is not possible (8). Nevertheless, other studies using MRI have reported changes in disc position after arthrocentesis (3,9).

The present study was carried out to determine whether arthrocentesis and hydraulic distension of the SJS is able to reduce the displaced disc in a woman with 6 weeks of painful closed lock due to DDwoR, and to assess the efficacy of such treatment in improving mouth opening and reducing joint pain.

## Case Report

**Figure 1:**
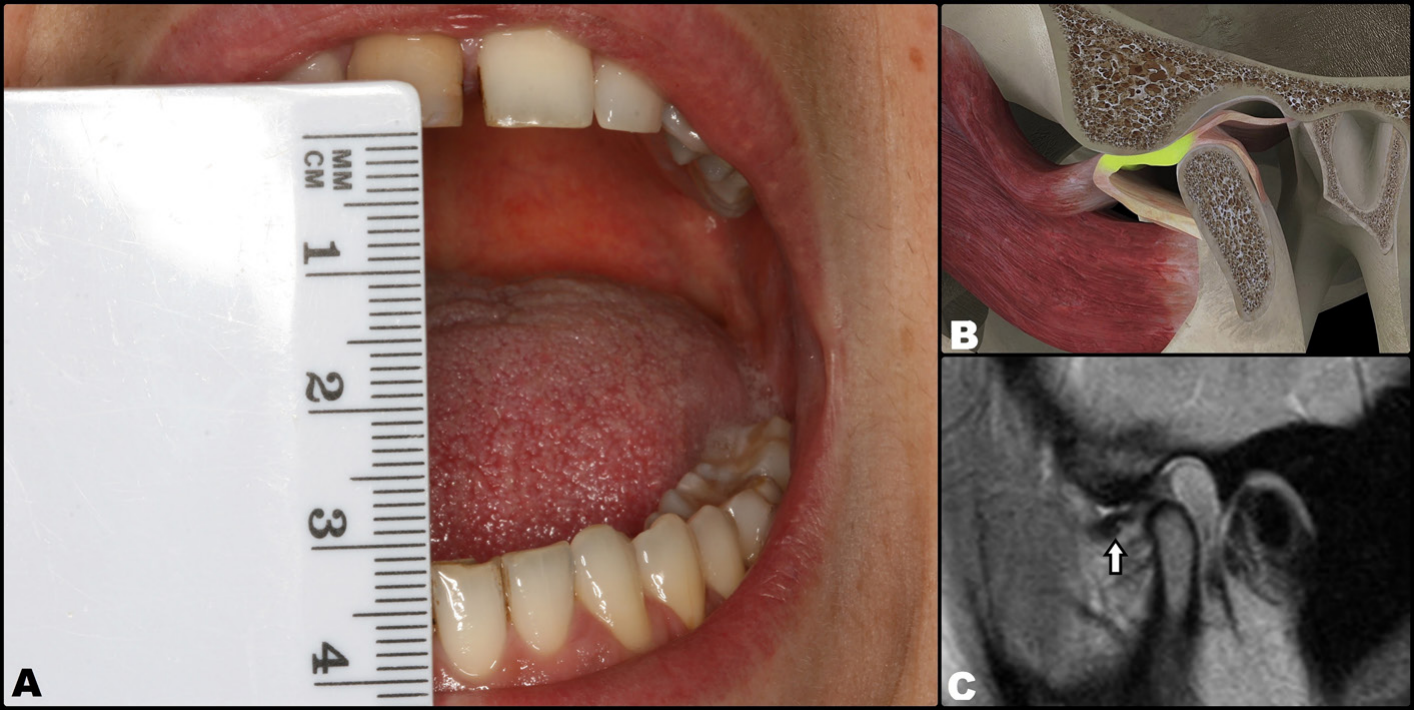
Initial situation. Disc displacement without reduction and with limited opening of the mouth of the patient. A: Opening limited to 30 mm. B: Three-dimensional representation at intra-articular level. C: Pre-treatment magnetic resonance imaging view (arrow: disc displacement).

A 34-year-old woman presented with a 6-week history of joint pain due to DDwoR of the right TMJ. The clinical manifestations consisted of moderately reduced mouth opening (30 mm) with deflection towards the affected side. MRI scan confirmed the diagnosis, showing anteromedial displacement of the joint disc with the mouth closed and no reduction at maximal mouth opening (MMO) (Fig. 1). This condition had been preceded in late 2019 by disc displacement with reduction (DDwR) and associated intense clicking sounds. Disc reduction through joint distraction (manual manipulation) proved unsuccessful.

**Figure 2:**
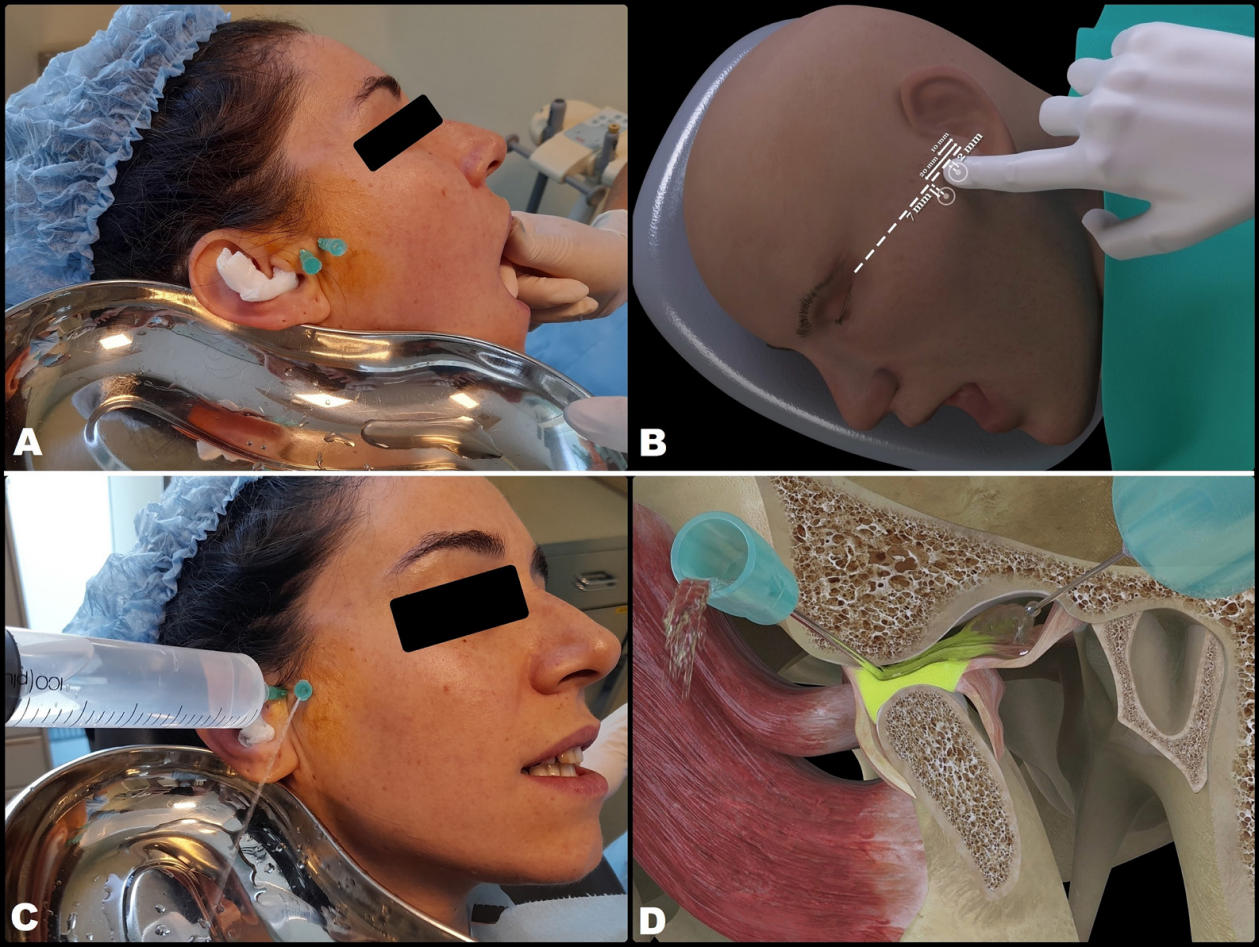
Arthrocentesis. A and B: Needle insertion sites. C and D: Intra-articular lavage and lysis.

**Figure 3:**
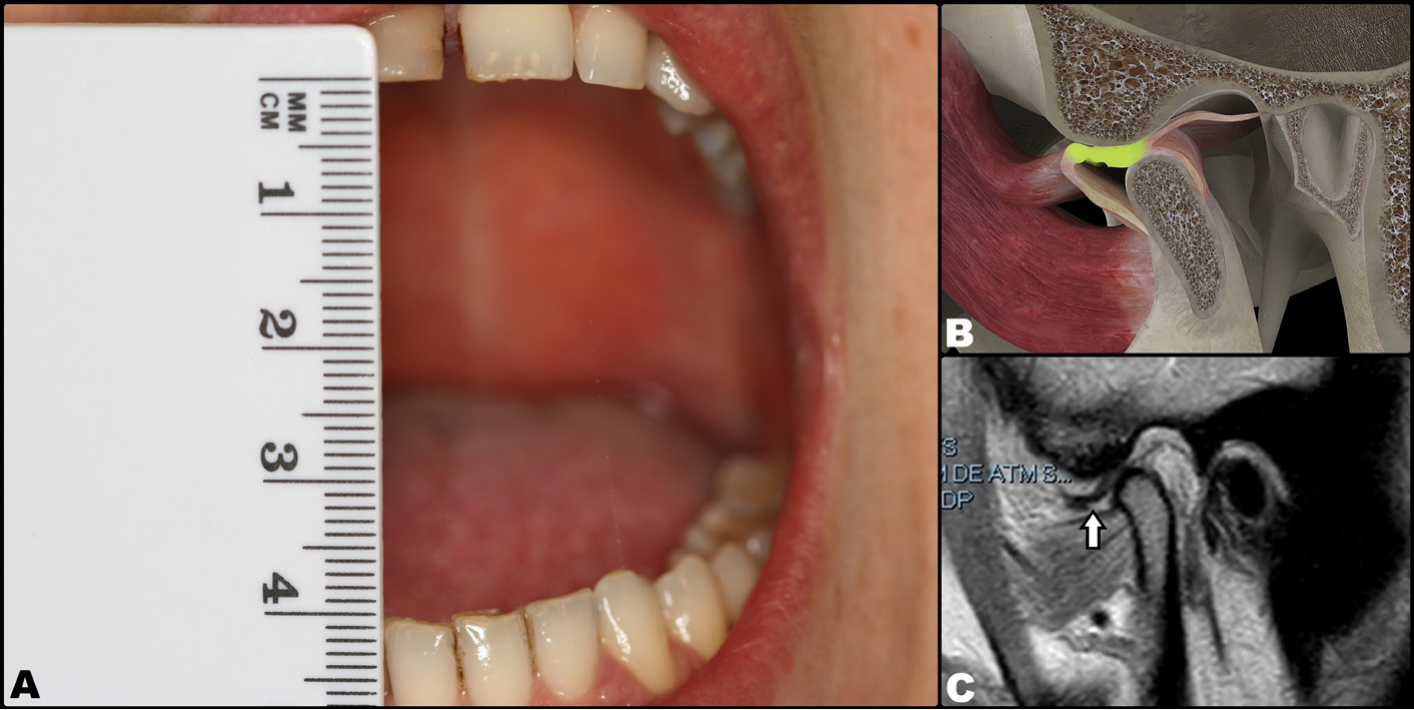
Final situation. Disc displacement without reduction and with no limited opening of the mouth of the patient. A: Correct opening reaching 40 mm. B: Three-dimensional representation at intra-articular level. C: Post-treatment magnetic resonance imaging view (arrow: disc displacement).

Arthrocentesis with the intra-articular infiltration of high molecular weight Hylan G-F 20 (a hyaluronic acid derivative) (Synvisc®, Genzyme Biosurgery, Ridgefield, NJ, USA) was proposed as treatment. The procedure was carried out under local anesthesia with articaine and epinephrine 1:200,000 (Ultracain®, Laboratorios Normon, Madrid, Spain), infiltrating the zone innervated by the auriculotemporal nerve in order to anesthetize the joint zone. Following anesthesia, MMO was seen to be 35 mm. Then, an intramuscular 21G needle measuring 25 mm in length was inserted into the SJS via the posterior recess under MMO of the patient. This needle was used to inject the solution used for lavage, while a second intramuscular 21G needle measuring 25 mm in length and positioned 5 mm anterior and inferior to the first needle was used to evacuate the solution (Fig. 2A,B). Joint lavage was performed with 120 ml of Ringer lactate (Braun, Barcelona, Spain). During lavage, evacuation of the solution was momentarily paused with a finger in order to increase intra-articular distension, reducing its negative pressure. The first 60 ml were used with the mouth of the patient open, and the remaining 60 ml were administered while the patient performed active opening, closing and lateralization movements (Fig. 2 C,D). At the end of the procedure, 1 ml of Hylan G-F 20 was injected as a cartilage-protecting measure thanks to its analgesic, lubricating and antiinflammatory effects. The result was excellent, with instantaneous and complete elimination of the joint closed lock, MMO of 40 mm, improvement of the mandibular movements, and disappearance of the joint pain. However, no clicks suggestive of having regressed to DDwR were noted. This situation persisted at the last control after three months, where the MRI scan showed the disc to remain displaced with MMO (Fig. 3).

## Discussion

Our question contemplated the possibility of reducing the joint disc during MMO of the patient following arthrocentesis, not only eliminating the joint pain and limitation of condylar displacement due to closed lock, since this can be achieved independently of the position of the disc (10), particularly if done in an early stage (8,11,12). Although some authors suggest that the morphological and organic changes of the TMJ with DDwoR are so severe that disc reduction is not possible (8), other investigators using MRI have reported changes in disc position after arthrocentesis (3,9).

Emshoff *et al*. (1) studied the MRI scans in a group of patients and found arthrocentesis to be a valuable tool for alleviating or eliminating joint pain and dysfunction, and for restoring MMO, though it did not modify the position or shape of the joint disc. However, these same authors subsequently refuted their previous findings (3), and reported that changes in disc position after arthrocentesis are possible, though with no statistically significant differences between the pre- and post-treatment MRI findings. These results are consistent with those published by Ohnuki *et al*. (9), who found that 1 of every 9 joints (11.1%) with DDwoR and subjected to arthrocentesis showed DDwR in the postoperative MRI study. However, they did not specify the duration of these joint closed locks changes.

There is controversy regarding the terminology referred to the duration of joint lock, with the term “acute” being used even for a duration of 6 months (13). However, based on the results of studies of arthrocentesis according to the duration of joint lock, we believe that this term should be modified, as already proposed by other authors (6,14).

Sembronio *et al*. (14) considered the term “acute” joint lock due to DDwoR to refer to limitation MMO (< 35 mm) for less than 4 weeks, this being the period in which there are greater chances for achieving disc reduction with MMO after arthrocentesis. These authors used MRI to determine whether the disc could be reduced in patients with DDwoR, and found that reduction proved possible in 62.5% of the “acute” cases. It should be noted that “recapturing” and repositioning the disc in its normal position with the mouth closed was even achieved in 37.5% of the cases (14).

On the other hand, Murakami *et al*. (6), in a study on the short-term effect of arthrocentesis in 20 joints with DDwoR and a mean evolution of 5.64 months, recorded a clinical failure rate of 30%; of these, almost 85% corresponded to cases of joint lock duration for over 7 months. It also should be mentioned that the mean age of the patients in which arthrocentesis failed was 39.3 years, compared to 27.6 years among the cases in which treatment proved successful, suggesting that elasticity of the superior retrodiscal lamina is lost earlier with age. Another study involving 40 cases of arthrocentesis published by Frost *et al*. (15) found that closed lock due to DDwoR and without sounds became a joint with opening and closing sounds (reciprocal clicks) consistent with DDwR after arthrocentesis. However, in a previous study by Murakami *et al*. (4) involving 10 patients, mean age 28.9 years, with “persistent” closed locks (mean duration 4.7 months) treated by “pumping” (hydraulic distension) and mandibular manipulation, although the authors managed to resolve the closed locks in all cases, only in 1 case of 7 months of joint lock did they confirm by arthroscopy that even the displaced disc was reduced with normal mouth opening.

In addition to statistical significance, which is important for the scientific validness of a study, we should also consider the conditions that favor disc reduction. The reduction of a displaced disc with a normal MMO seems to be possible, though it depends on the duration of closed lock (4,6,14,15). Thus, in view of the results of the studies found in the literature, we prefer to use the term “acute” for a duration of lock of less than 1 month, with “subacute” being used in reference to a duration of 1-6 months, and “chronic” to a duration of over 6 months. We deduce that in cases of “acute” lock, many discs can be recaptured, while in “subacute” cases the recapture rate is low, and in “chronic” cases recapture is practically impossible. It may be speculated that over time, the fibers of the superior retrodiscal laminas lose their elasticity and tend to suffer fibrosis, causing the retraction and reduction of displaced disc to occur further over the trajectory of anterior condylar translation, reaching a point where disc deformation becomes so important that “self-positioning” on the condyle in its intermediate region is not possible. We believe that this hypothesis could explain why disc reduction can be achieved in many “acute” cases.

Although a disc reduction favors a larger MMO since it allows the condyle to move forward along its entire natural path, it has been shown that in cases of disc displacements, if the condyle is allowed to push forward, it deforms and the retrodiscal tissue elongates, so that the path is practically complete and clinically there is no limitation of the mouth opening (16). Although the mandibular condyle is located under the retrodiscal tissues, the latter adapt over time with connective tissue changes, finally acting as a “pseudodisc” (17). In order to achieve disc deformation and elongation of the retrodiscal tissues, early disc mobilization is essential, since its immobilization caused by the joint pain impairs synovial lubrication, and in advanced cases produces adherences with a negative impact upon patient quality of life due to chronic limitation of mouth opening, with the ulterior need for more invasive treatments such as arthroscopy (11,12). Thus, the main aim of arthrocentesis should be to eliminate joint pain as soon as possible in order to favor mandibular movement (18).

## Conclusions

Joint disc reduction in closed lock is possible in some cases if treatment is applied early, but is not essential for the management of these patients, since arthrocentesis allows disc mobilization and deformation impelled by the mandibular condyle, with resolution of the limitation of mouth opening. The main objective of arthrocentesis should be to eliminate the pain as soon as possible in order to allow mobility of the mandible and, with it, that of the joint disc.

## Data Availability

The datasets used and/or analyzed during the current study are available from the corresponding author.
